# 

**Published:** 1880-07-20

**Authors:** 


*Receipts* will appear in our next.

☞Subscribers who have not done so will please send up their dues.

*College Notices.*—Read the advertisement of the Medical College at New Orleans in the present issue.

☞See advertisement of *Southern Midical College*, Atlanta, Ga. In writing to this Institution address Dr. R. C. Word, Dean, as there are three schools in the city, and mistakes are frequently made.

*Three Terms.*—The American Medical College Association have adopted the three terms requirement to take effect at the session of 1882. And most if not all the schools, it is supposed, will adopt the measure.

